# Evidence for the cholinergic markers ChAT and vAChT in sensory cells of the developing antennal nervous system of the desert locust *Schistocerca gregaria*

**DOI:** 10.1007/s10158-020-00252-4

**Published:** 2020-10-22

**Authors:** Erica Ehrhardt, George Boyan

**Affiliations:** 1 Graduate School of Systemic Neuroscience, Biocenter, Ludwig-Maximilians-Universität München, Grosshadernerstrasse 2, 82152 Planegg, Martinsried, Germany; 2 Institute of Zoology, Universität Köln, Zülpicher Str 47b, 50674 Cologne, Germany

**Keywords:** Locust, Antenna, Development, Sensory cells, ChAT, vAChT

## Abstract

Sensory and motor systems in insects with hemimetabolous development must be ready to mediate adaptive behavior directly on hatching from the egg. For the desert locust *S. gregaria*, cholinergic transmission from antennal sensillae to olfactory or mechanosensory centers in the brain requires that choline acetyltransferase (ChAT) and the vesicular acetylcholine transporter (vAChT) already be present in sensory cells in the first instar. In this study, we used immunolabeling to demonstrate that ChAT and vAChT are both expressed in sensory cells from identifiable sensilla types in the immature antennal nervous system. We observed ChAT expression in dendrites, neurites and somata of putative basiconic-type sensillae at the first instar stage. We also detected vAChT in the sensory axons of these sensillae in a major antennal nerve tract. We then examined whether evidence for cholinergic transmission is present during embryogenesis. Immunolabeling confirms that vAChT is expressed in somata typical of campaniform sensillae, as well as in small sensory cell clusters typically associated with either a large basiconic or coeloconic sensilla, at 99% of embryogenesis. The vAChT is also expressed in the somata of these sensilla types in multiple antennal regions at 90% of embryogenesis, but not at earlier (70%) embryonic stages. Neuromodulators are known to appear late in embryogenesis in neurons of the locust central complex, and the cholinergic system of the antenna may also only reach maturity shortly before hatching.

## Introduction

Acetylcholine is a ubiquitous neuromodulator with extensive roles in insect physiology and behavior (Heinrich et al. 1997; Kunst et al. 2011; Boppana et al. 2017; Deshpande et al. 2020; Showell et al. 2020). In the fly, for example, most chemosensory, olfactory, chordotonal, and auditory primary sensory neurons are cholinergic (Salvaterra and Kitamoto 2001). In the locust, components of cholinergic transmission including acetylcholinesterase, choline acetyltransferase, the high affinity choline transport system, and acetylcholine receptors are expressed throughout the adult CNS (see Homberg 2002 for review). The central projections of antennal afferents in the brain are also cholinergic (Knipper et al. 1989; Rind and Leitinger 2000), although the sensilla types involved remain unidentified.

In insects with a hemimetabolous mode of development, sensory and motor systems must contribute to adaptive behavior directly on hatching from the egg (Stevenson and Kutsch 1986). Cholinergic transmission in the antennal nervous system of the desert locust *S. gregaria*, for example, must therefore be functional at this first instar stage for olfactory and mechanosensory information to reach regulatory centers in the brain (Gewecke 1972). Despite this, developmental data on cholinergic neurotransmission in this sensory system are lacking.

In this study, we use immunolabeling to demonstrate that two essential components of a functional cholinergic system—choline acetyltransferase (ChAT) which catalyzes the synthesis of acetylcholine and the vesicular acetylcholine transporter (vAChT) which packs the transmitter into vesicles for synaptic release (see Deshpande et al. 2020) are present in sensory cells of the locust antenna at this first instar stage. We propose identities for the sensilla types involved and also demonstrate that vAChT expression in these cells commences during embryogenesis, but only during the later stages (after 90%), suggesting that the cholinergic system of the antenna may only reach maturity shortly before hatching.

## Materials and methods

Eggs from a crowded colony of *Schistocerca gregaria* were maintained as described in Ehrhardt et al. (2015, 2016) and embryos staged according to Bentley et al. (1979). Prior to immunolabeling, preparations were either embedded and sectioned (14 µm), or treated with ultrasound (sonication) to render the cuticle porous and allow imaging of the intact antenna (for details see Ehrhardt et al. 2015, 2016).

### Immunolabeling

For protocols detailing solutions and incubation conditions, see Ehrhardt et al. (2015, 2016).

#### Primary antibodies

*ChAT* the polyclonal antibody against mammalian choline acetyltransferase (α-ChAT, rabbit, Millipore AB143, dilution 1:100 in incubation medium) labels neuronal somata, dendrites and axons in the locust (see also Geffard et al. 1985; Lutz and Tyrer 1987).

*vAChT* the polyclonal antibody against the mammalian vesicular acetylcholine transporter (α-vAChT, guinea pig, Millipore AB 1588, dilution 1:500) labels auditory sensory cells in bushcrickets (Weber et al. 2005).

*HRP* the antibody against horseradish peroxidase (HRP) recognizes a neuron-specific cell surface epitope in insects (Jan and Jan 1982). In single-labeling experiments, we used: polyclonal α-HRP (Dianova, rabbit, 323-005-021, dilution 1:150). For double-labeling experiments with α-ChAT, we used polyclonal α-HRP (Dianova, goat, 123-005-021, dilution 1:150), and for experiments with α-vAChT, we used polyclonal α-HRP (Dianova, rabbit, 323-005-021, dilution 1:150).

#### Secondary antibodies

For single-labeling experiments with HRP, we used Alexa® 488 (goat anti-rabbit, Invitrogen A11034) or Cy3 (goat anti-rabbit, Dianova 111-165-003) each diluted 1:200. For double-labeling experiments, the combinations were α-ChAT (Cy3, donkey anti-rabbit, Dianova 711-167-003, dilution 1:200)/α-HRP (Alexa® 488, donkey anti-goat, Dianova 705-547-003, dilution 1:450) and α-vAChT (Alexa® 488, goat anti-guinea pig, Invitrogen A 11073, dilution 1:150)/α-HRP (Cy3, goat anti-rabbit, Dianova 111-165-003, dilution 1:150).

### Imaging

Confocal and fluorescence microscopy was performed as previously described (Boyan et al. 2010; Ehrhardt et al. 2015, 2016). ImageJ software (public domain) was used for all image processing and involved adjusting only contrast and resolution, the allocation of false colors, and the application of a convolution algorithm on selected fluorescence photomicrographs.

## Results

Our results confirm the presence of the cholinergic markers choline acetyltransferase (ChAT) and the vesicular acetylcholine transporter (vAChT) in sensory cells of the developing antenna of the desert locust *S. gregaria*.

### Sensory cell clusters of the flagellum

**Fig. 1:**
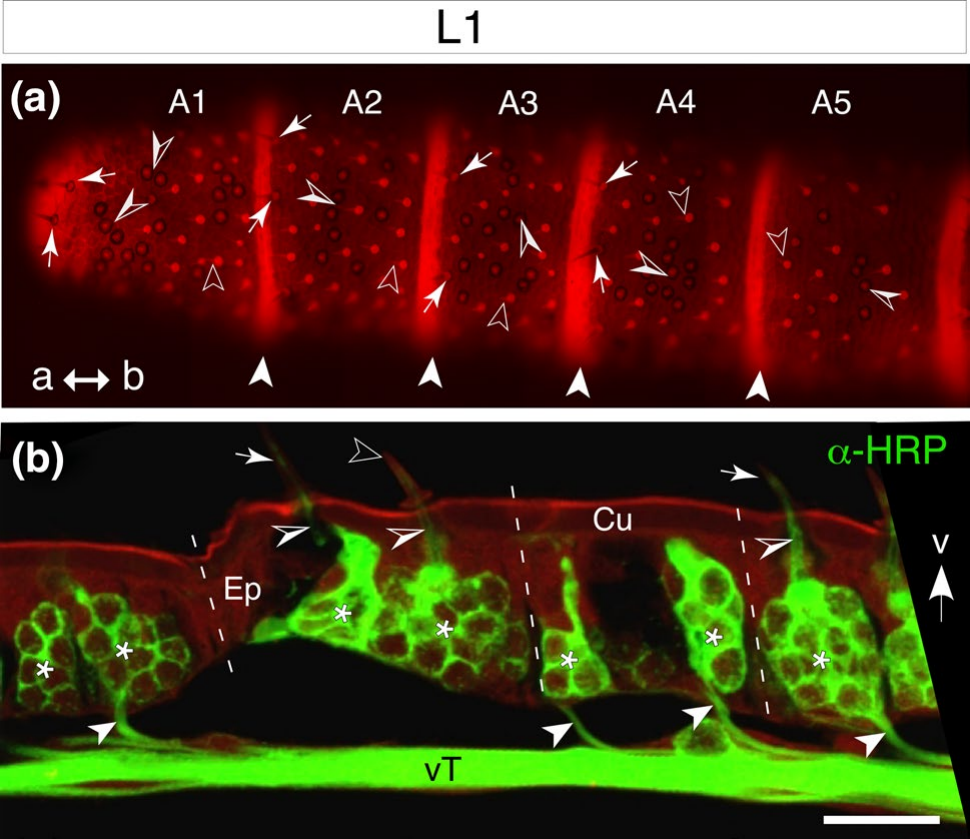
Sensillae and sensory cell clusters of the locust antenna at the 1st instar stage (L1). **a** Fluorescence photomicrograph of the ventral surface of the antenna shows autofluorescent (red) septal-like cuticular restrictions (white arrowheads) delimiting the five apical-most meristal annuli (A1–A5) of the flagellum. Large basiconic (white arrows), short basiconic (open white arrowheads) and coeloconic (white/open arrowheads) sensillae can be identified (see Slifer et al. 1959; Chapman and Greenwood 1986; Ochieng et al. 1998). Coordinates point to apex (a) and base (b) of the antenna for both panels. **b** Confocal image of a longitudinal section through the antenna following immunolabeling with neuron-specific anti-horseradish peroxidase (HRP, green). White arrow points to ventral (v) cuticular surface. Clusters of neuronal somata (white asterisks) are visible in four meristal annuli of the flagellum (white dashed lines indicate cuticular restrictions). Sensory dendrites (open/white arrowheads) innervate large basiconic (white arrows), or short basiconic (open white arrowheads) sensillae on the ventral surface. Cells from each cluster direct bundled axons (white arrowheads) to the ventral antennal nerve tract (vT). Scale bar represents 135 µm in (**a**), 30 µm in (**b**) (color figure online)

On hatching (1st instar stage), the flagellum of the locust comprises 11 segments known as meristal annuli (A1–A11, see Chapman 2002) each delimited by septal-like cuticular restrictions (Fig. 1a). Large numbers of basiconic, coeloconic and campaniform sensillae cover the antennal surfaces (see Slifer et al. 1959; Chapman and Greenwood 1986; Ochieng et al. 1998 for details). Depending on the type, these sensillae are innervated by one or more dendrites originating from HRP-positive sensory cells located at the base, whose axons project via either a ventral (vT) or dorsal (dT) nerve tract to the brain (Fig. 1b).

### ChAT in sensory neurons

**Fig. 2:**
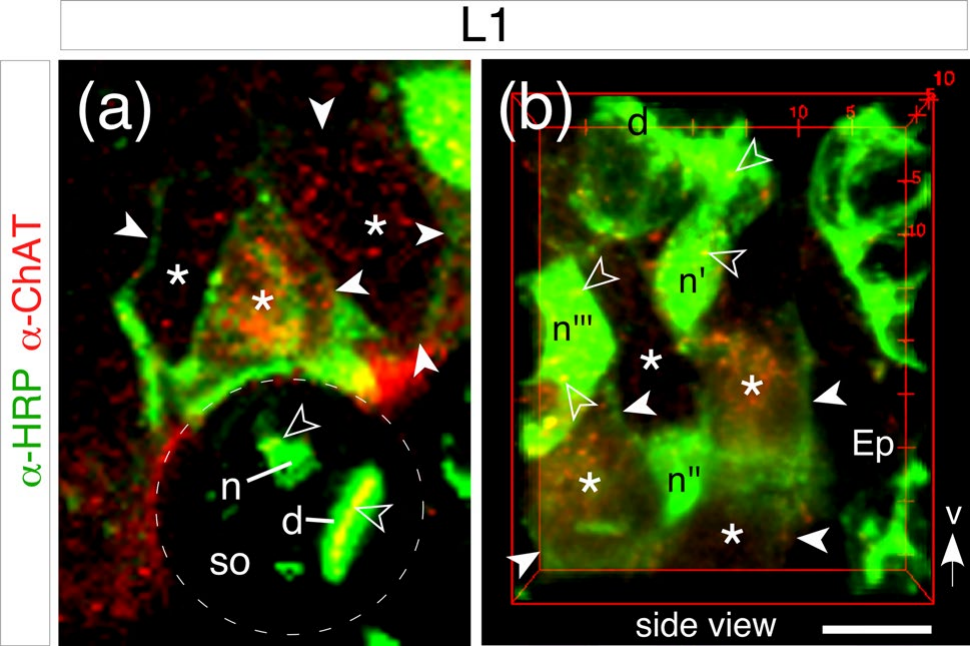
ChAT is expressed in antennal sensory neurons at the 1st instar stage (L1). Preparations were sonicated (see Methods) prior to double-labeling with α-HRP (green) and α-ChAT (red). **a** Confocal image shows the outline (dashed white) of the sensillar socket (so), with an HRP-positive dendrite (d) projecting into the sensilla tip, a bulge at the sensilla base indicates the beginning of the neurite (n) which runs (open white arrowheads) deeper into the antenna to a small cluster of HRP-positive sensory cells (white arrowheads, green). The morphological features are consistent with those of a large basiconic hair (see Slifer et al. 1959) and have not been disrupted by sonication. ChAT (red) is present in somata (white asterisks) and is co-localized (yellow) to the dendrite (d) and neurite (n). **b** 3D confocal image within a bounding box shows the organization of a basiconic-type sensilla from a second antenna but in side view following double-labeling with α-HRP (green) and α-ChAT (red). White arrow points toward ventral cuticular surface (v). An HRP-positive dendrite (d) from the sensilla (not in view) projects from the cuticular surface to the sensilla base and joins the bulge of a neurite (n) which then extends deeper (downward) within the epithelium (Ep) to a cluster of HRP-positive (white arrowheads) sensory cells. Somata (white asterisks, red) and neurites (open white arrowheads, yellow) are positive for ChAT. Scale bar represents 10 µm (color figure online)

Double-labeling experiments against neuron-specific horseradish peroxidase (α-HRP) and anti-choline acetyltransferase (α-ChAT) demonstrate the presence of ChAT in HRP-positive sensory neurons in sonicated first instar antennae (Fig. 2a, b). Morphological features such as the bulge in the neurite at the sensilla base (Fig. 2b) are consistent with those of basiconic-type sensillae (c.f. Slifer et al. 1959) and are intact following sonication (see Methods here and Ehrhardt et al. 2015). ChAT also appears to be present in the dendrite (Fig. 2a) and neurites (Fig. 2b) of these sensory units.

**Fig. 3:**
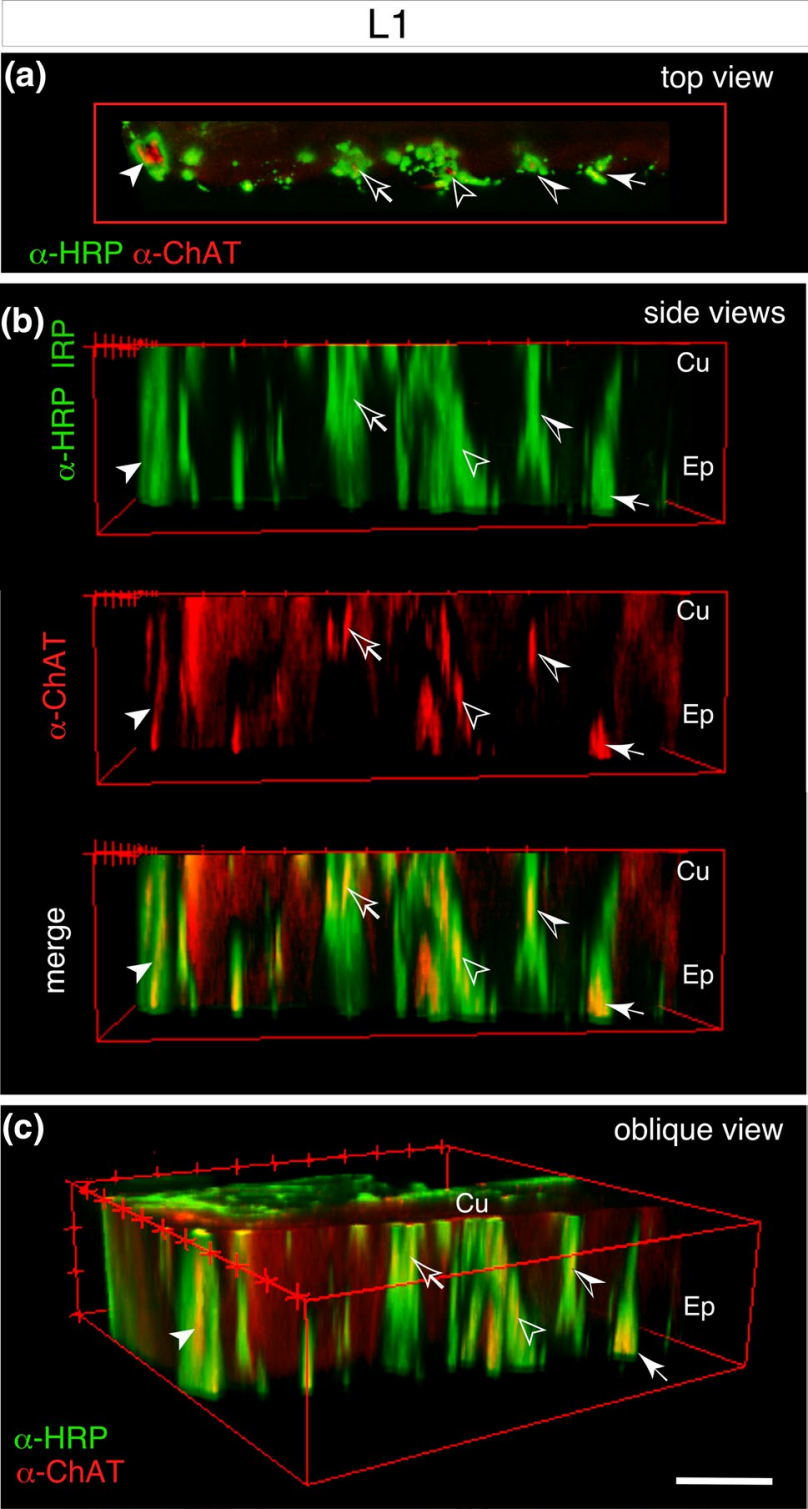
Evidence for ChAT in neurites of antennal sensory cells at the first instar stage (L1). **a** Confocal image taken just below the cuticular surface (viewed from the top) following sonication and then double-labeling against neuron-specific HRP (α-HRP, green) and ChAT (α-ChAT, red). HRP-positive neurites from clusters of sensory cells also test positive for ChAT. Each cluster is marked by a unique character which is consistent from panel to panel. **b** Reconstructions (side views) of the confocal stack from panel a shows HRP-positive (green) and ChAT-positive (red) neurites belonging to the cell clusters identified in panel a and extending from just below the cuticular surface (Cu) deeper into the epithelium (Ep) toward sensory somata (not in view). Superposition of images (merge) confirms co-localization (yellow) of ChAT to HRP-positive neurites. **c** 3D reconstruction of the confocal stack from panel **b** rotated obliquely (bounding box indicates extent of rotation) reveals co-localization (yellow) of ChAT (red) to HRP-positive neurites (green) which extend through the epithelium (Ep) from just below the cuticular surface (Cu). Scale bar represents 20 µm (color figure online)

Confirmation that neurites contain ChAT was obtained from 3D reconstructions following double-labeling (α-HRP, α-ChAT) in sonicated antennae (Fig. 3). Confocal imaging of a preparation viewed at a level just below the cuticle (Fig. 3a, top view) reveals clusters of HRP-positive neurites in each of which ChAT-labeling is visible. 3D confocal imaging through the antenna and subsequent rotation of the optical stack to either a side (Fig. 3b) or oblique (Fig. 3c) view confirms ChAT labeling to be co-localized to neurites which run from the cuticular surface deeper into the epithelium toward their sensory somata (not in view).

### The vAChT in sensory axons

**Fig. 4:**
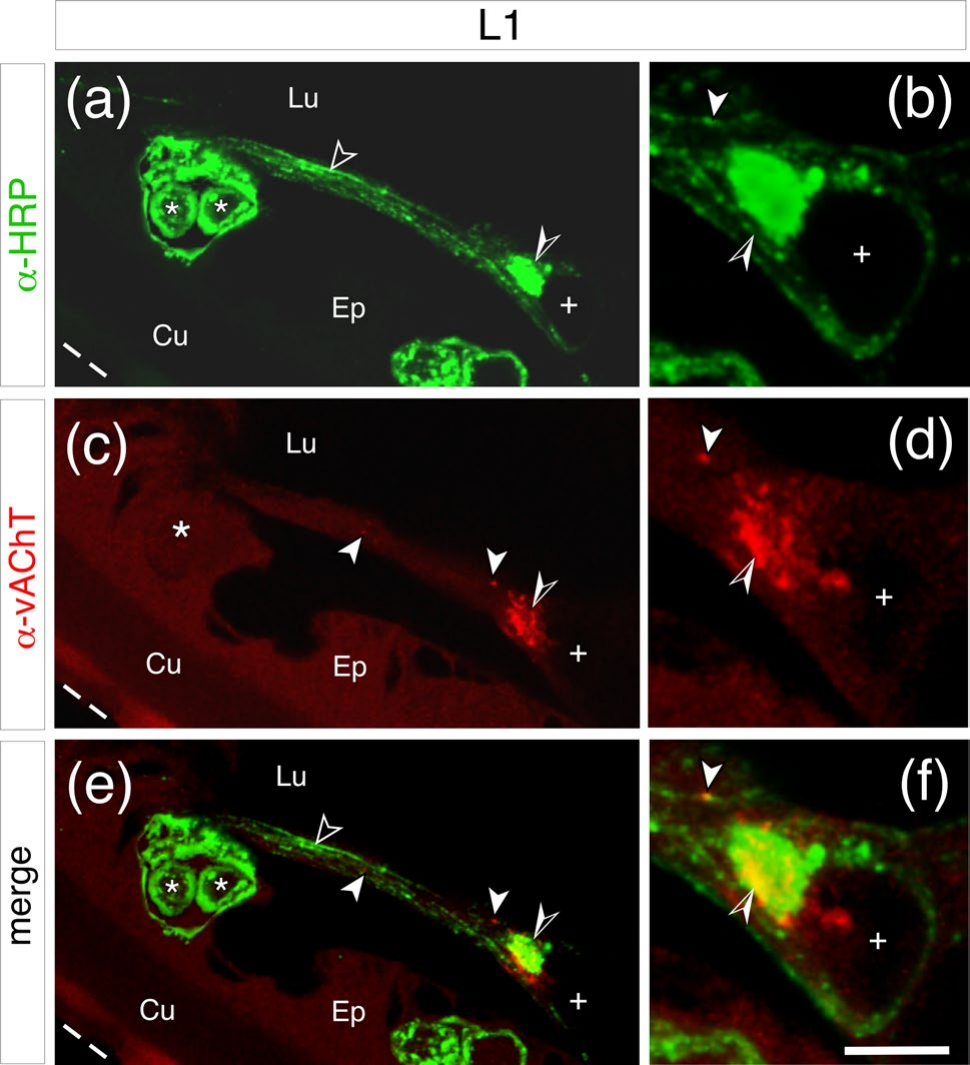
Evidence for the vesicular acetylcholine transporter (vAChT) in sensory axons of the 1st instar antenna (L1). Confocal images are from a longitudinal section through the antenna following double-labeling with neuron-specific α-HRP (green) and α-vAChT (red). The antennal apex is toward the top left, outer edge of the cuticle (Cu) is dashed white. **a** A subset of HRP-positive sensory cells (white asterisks) in the epithelium (Ep) projects axons (open white arrowhead) in a peripheral nerve within the lumen (Lu) toward the antennal base. En route, sensory axons form a plexus (open/white arrowhead) with a further HRP-positive neuron (white cross) seen at higher magnification in (**b**). **c** Immunolabeling demonstrates the presence of vAChT (red) in the sensory axons (white arrowheads) and the plexus region (open/white arrowhead) seen at higher magnification in (**d**). **e** Superposition (merge) confirms the co-localization (yellow) of vAChT to the HRP-positive sensory axons (white arrowheads) from the sensory cell cluster (white asterisks), the plexus region (open/white arrowhead) and the unidentified neuron (white cross) seen at higher magnification in (**f**). Scale bar represents 25 µm in (**a**, **c**, **e**); 15 µm in (**b**, **d**, **f**) (color figure online)

The presence of the vesicular acetylcholine transporter (vAChT) in sensory axons would be consistent with cholinergic synaptic transmission in the antennal nervous system already at the first instar stage. Double-labeling (α-HRP, α-vAChT) after longitudinal sectioning at this first instar stage revealed an HRP-positive sensory cell cluster (white asterisks, Fig. 4a) from which bundled axons project within a nerve tract toward the antennal base (open arrowhead, Fig. 4a). En route these axons appear to form a plexus with a further, unidentified, HRP-positive neuron (open/white arrowhead, Fig. 4b). Examination of the vAChT channel (Fig. 4c, d) confirms the presence of the vAChT, which superposition (Fig. 4e, f) shows, is co-localized to both the sensory axons in the nerve tract (white arrowhead), their terminals in the plexus region (open/white arrowhead) and the putative target neuron (white cross).

### The vAChT in the embryonic antenna

If components of a cholinergic neuromodulatory system such as ChAT (Figs. 2 and 3) and the vAChT (Fig. 4) are already present in sensory units of the antenna on hatching, then it is more than likely that these have developed earlier, during embryogenesis. In the course of this study, we found that the signal strength using α-vAChT was consistently clearer and stronger than with α-ChAT (this may be a methodological problem and needs to be addressed in future studies). As a consequence, we reasoned that to test for, and interpret, any developmentally based changes in expression we would need the strongest signal to begin with, and this was with α-vAChT.

**Fig. 5:**
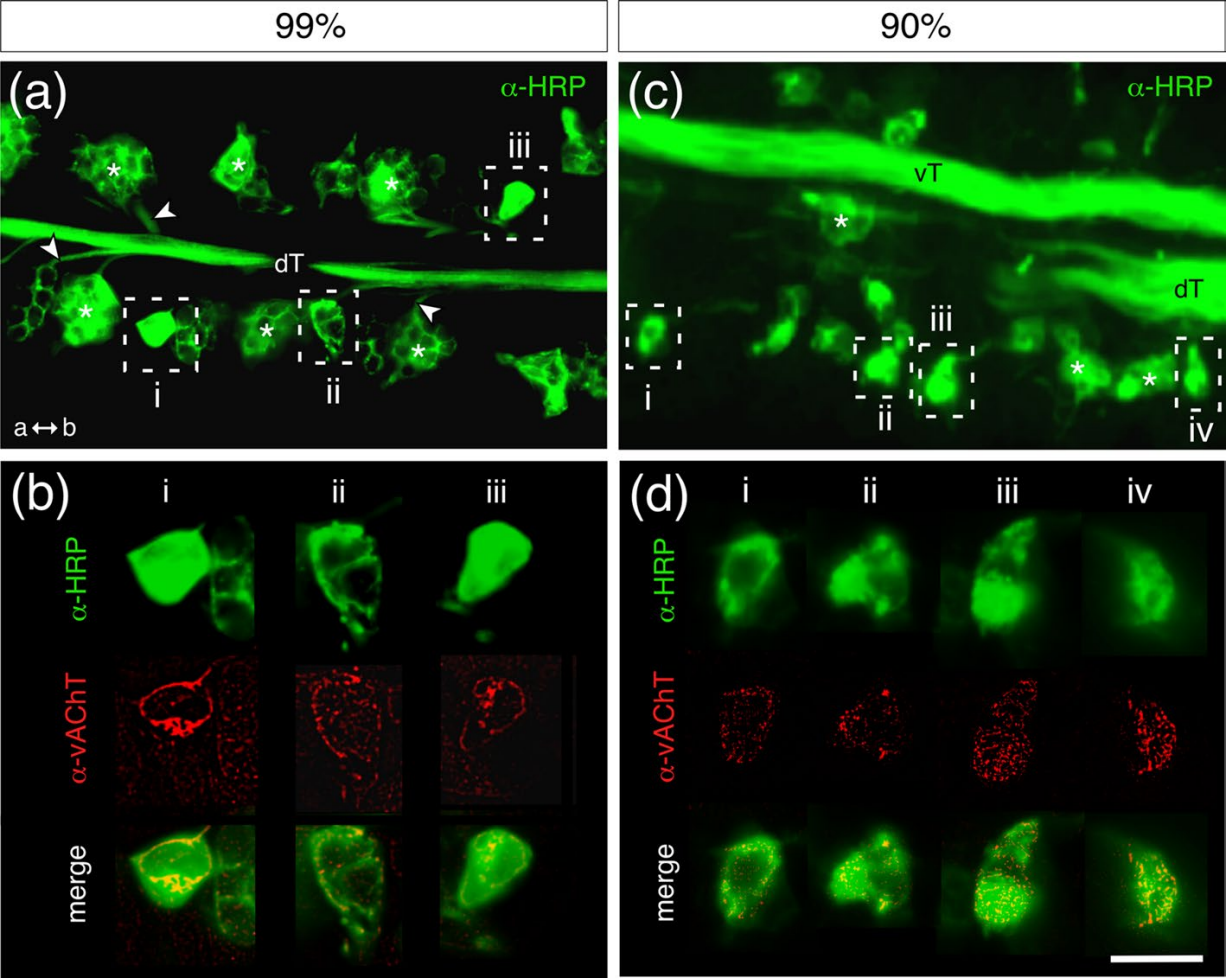
Evidence for the vAChT in sensory cells of the antenna during embryogenesis. **a** Fluorescence photomicrograph of a longitudinal section through the antenna following labeling against neuron-specific HRP (green) at 99% of embryogenesis. Clusters of sensory somata (white asterisks) direct axons (white arrowheads) to the dorsal antennal nerve tract (dT). Axes point to antennal apex (a) and base (b) here and in panel. **c** HRP-positive sensory somata from three regions (white dashed rectangles labeled i–iii) are shown at higher magnification in panel **b** following co-labeling with α-vAChT (red). Images for α-vAChT here and in panel **d** follow convolution with ImageJ (see Methods). Superposition of images (merge) confirms the co-localization (yellow) of vAChT to the HRP-positive sensory somata in all three regions. **c** Fluorescence photomicrograph of a longitudinal section through the antenna following labeling with α-HRP (green) at 90% of embryogenesis shows clusters of sensory somata (white asterisks) and the ventral (vT) and dorsal (dT) nerve tracts. HRP-positive sensory somata from four regions (white dashed rectangles labeled i–iv) are shown at higher magnification in panel **d** following co-labeling with α-vAChT (red). Superposition of images (merge) confirms the co-localization (yellow) of vAChT to the HRP-positive sensory somata from all four regions. Scale bar represents 30 µm in (**a**, **c**); 15 µm in (**b**, **d**) (color figure online)

We therefore first double-labeled (α-HRP, α-vAChT) sectioned antennae from preparations just prior to hatching (99%, Fig. 5a, b). Large HRP-positive individual somata typical of campaniform sensillae (Fig. 5a, b: i, iii), as well as small HRP-positive sensory cell clusters (Fig. 5a, b: ii) typically associated with either a large basiconic or coeloconic sensilla (c.f. Slifer et al. 1959), direct axons to the dorsal antennal nerve tract (dT, Fig. 5a). Both neuron types also express vAChT which co-localizes to the sensory somata from sensillae of all three regions examined at this developmental stage (Fig. 5b).

We then investigated younger antennae (90% of embryogenesis) for evidence of the vAChT (Fig. 5c, d). The HRP-positive ventral (vT) and dorsal (dT) antennal nerve tracts are visible in the section, along with clusters of HRP-positive sensory somata whose processes project to these tracts. Somata of putative basiconic or coeloconic sensillae selected from four regions of the antenna are vAChT-positive (Fig. 5d).

We were unable to document the vAChT in the antennal sensory system at a still earlier embryonic stage (70%).

## Discussion

**Fig. 6:**
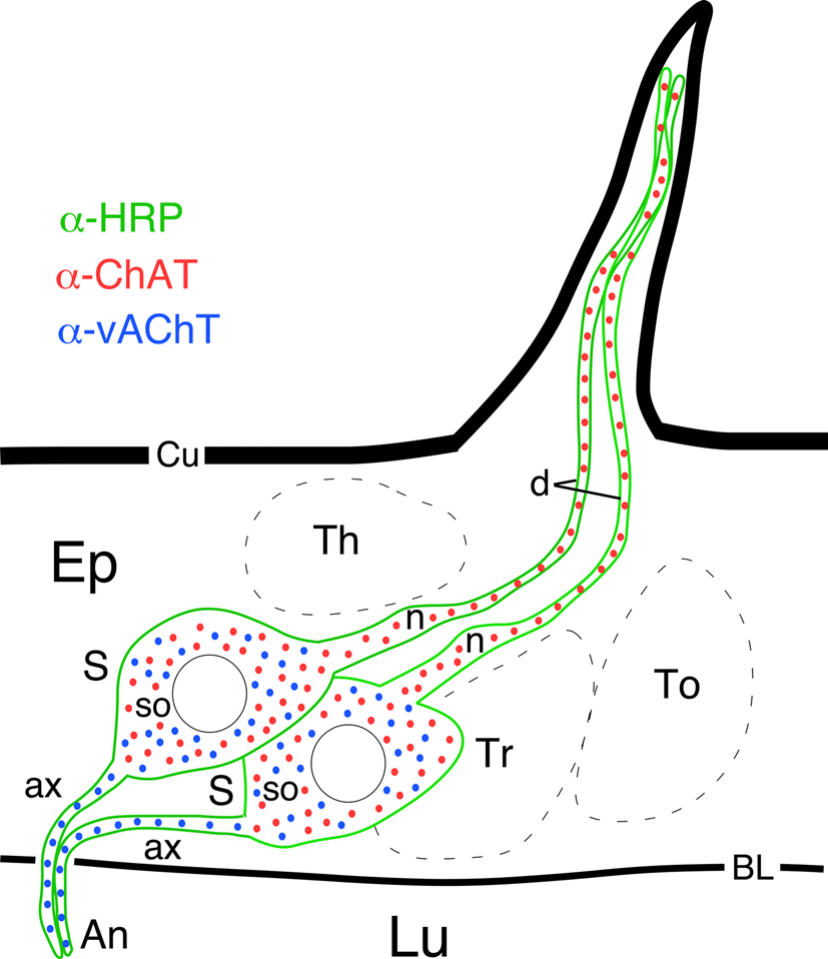
Schematic (not to scale) illustrates the distribution of the cholinergic markers ChAT (red) and vAChT (blue) in the HRP-positive (green) sensory cells of a basiconic-type sensillum on the antenna of the locust. Data cover the developmental stages from 90% of embryogenesis to the 1st postembryonic instar. The schematic is representative and not intended to be anatomically or functionally complete, showing only some of the basic cellular elements belonging to such a sensory unit within the epithelium (Ep) (see Slifer et al. 1959 for definitive description). ChAT expression was found in the soma (so), neurite (n) and dendrite (d) of each sensory cell (S); vAChT was located in the somata (so) and axons (ax) which run via an antennal nerve (An) in the lumen (Lu) to the brain. Cell types not involved in this study are the thecogen (Th), tormogen (To) and trichogen (Tr) cells. Other abbreviations: *BL* basal lamina, *Cu* outer cuticle (color figure online)

In this study, we focused on the expression of two components of cholinergic transmission in the developing antennal nervous system of the desert locust *S. gregaria*—choline acetyltransferase (ChAT) which catalyzes the synthesis of acetylcholine, and the vesicular acetylcholine transporter (vAChT) which packs the transmitter into vesicles for synaptic release. Our results cover the developmental stages from 90% of embryogenesis to the 1st postembryonic instar and are summarized schematically in Fig. 6.

### Evolutionary aspects

Classical transmitters are present in all phyla that have been studied (see Walker and Holden-Dye 1991 for a review). With respect to the cholinergic system, Banzai et al. (2015) show that both choline acetyltransferase (ChAT) and the vesicular acetylcholine transporter (vAChT) are conserved across phyla. Their comparisons of ChAT-cDNA reveal about 64% sequence homology between *Bombyx mori, Drosophila*, and *Aedes*, while ChAT-cDNA from mouse has a 58% homology to that in *Bombyx mori*. Further, vAChT-cDNA is apparently even more highly conserved: there is a 78% sequence homology between *Bombyx* and *Drosophila*, while mouse vAChT-cDNA has a 70% homology to that in *Bombyx*.

In adult bushcrickets, Weber et al. (2005) used a mammalian polyclonal antibody to demonstrate that auditory sensory neurons in the forelegs express a vAChT corresponding to the molecular mass of *Drosophila* vAChT (c.f. Kitamoto et al. 1998), while Leitinger and Simmons (2000) also employed a mammalian polyclonal antibody to localize ChAT in neurons of the locust CNS and used a Western blot to confirm that the mammal ChAT antibody was specifically labeling a protein of the expected molecular weight in the locust. The high affinity obtained with the polyclonal antibodies against mammalian ChAT and vAChT we used for the immunolabeling experiments in this study (see “Materials and methods section) are further testimony to the conserved nature of these key cholinergic molecules.

### Acetylcholine in the insect sensory nervous system

While insect nervous systems express a wide range of neuromodulators (see Homberg 2002; Nässel 2002; Python and Stocker 2002; Deshpande et al. 2020), cholinergic transmission in particular features strongly in their chemosensory and mechanosensory subsystems (Lutz and Tyrer 1987; Rind and Simmons 1998; Leitinger and Simmons 2000; Knipper et al. 1989; Leitch et al. 1993; Hermsen et al. 1998; Python and Stocker 2002; Salvaterra and Kitamoto 2001). In the fly, for example, most chemosensory, olfactory, chordotonal and auditory primary sensory neurons are cholinergic (Salvaterra and Kitamoto 2001). In the adult locust too, central projections from the antennal nerve to the brain are also cholinergic (Knipper et al. 1989; Rind and Leitinger 2000) but the identity of the sensory sensillae involved remains unclear, and a timeline for the development of cholinergic expression in the antennal system is lacking.

### Choline acetyltransferase (ChAT)

We show that ChAT is present in the somata of sensory cells associated with basiconic-type sensillae on the antenna of the first instar locust (Figs. 2 and 6). The dendrites and neurites from these sensillae are also labeled (Figs. 2, 3 and 6), and this has a precedent in mammalian nervous systems where dendrites from pontomesencephalic neurons of tegmental nuclei in the cat are also known to express ChAT (Jia et al 2003). Our data are also consistent with findings from the fly, where ChAT is expressed in sensory neurons associated with basiconic, trichoid and coeloconic sensillae (Yasayama and Salvaterra 1999). While ChAT is found in chemosensory and proprioceptive sensory neurons of stage-15 fly embryos, it is not present in tactile bristles of the adult (Yasayama and Salvaterra 1999) which express histamine (Buchner et al. 1993), one of several neuromodulators reported for insect (Persson and Nässel 1999; Python and Stocker 2002) and spider (Fabian-Fine et al. 2017) mechanosensory pathways.

### The vesicular acetylcholine transporter (vAChT)

The vAChT (see Boppana et al. 2017; Deshpande et al. 2020 for reviews) not only facilitates quantal transmitter packing (Song et al. 1997), but in *Drosophila* also plays a role in glial differentiation (Soustelle et al. 2002), cognitive performance and locomotion (Showell et al. 2020). Given the homologous nature of insect nervous systems (see Meier and Reichert 1991; Meier et al. 1991; Boyan and Ball 1993; Casares and Mann 1998), we expected to find the vAChT in the developing locust antennal nervous system as well. The vAChT was present in HRP-positive axons from antennal cell clusters of the first instar locust (Figs. 4 and 6). Further, our data show that en route to the brain these sensory axons form a plexus with at least one other HRP/vAChT-positive cell (Figs. 4b, d, f and 6) which leads us to speculate that presynaptic interactions similar to those reported for the cercal nervous systems of the first instar cockroach (Blagburn and Sattelle 1987) and adult locust (Boyan 1988) may also contribute to sensory processing in the locust antennal system.

### Developmental aspects

The presence of the vAChT in the antennal nervous system of the first instar locust suggests that cholinergic transmission develops earlier, that is during embryogenesis. We show that the vAChT is immunohistochemically detectable in the somata of antennal sensory cells typical of campaniform, large basiconic or coeloconic antennal sensillae at 99% (Figs. 5a, and 6) and 90% (Figs. 5b, and 6) of embryogenesis. We failed to detect vAChT at still earlier stages (70%), and it is possible that the vAChT is expressed in younger embryos but remains below the detection limits of our protocols. During metamorphosis of the moth antennal system, levels of ChAT, ACh and AChE expression only increase significantly at metamorphosis as more and more neurons differentiate (Sanes and Hildebrand 1976; Sanes et al. 1977). Our data, however, are consistent with other studies in the embryonic locust which report that while receptors for neuromodulators develop quite early (Goodman and Spitzer 1980), the synthesis of the neuromodulators themselves commences considerably later in the central nervous system (Goodman et al. 1979) and follows a programmed timeline unique to each neuromodulator (Thompson and Siegler 1991). In neuronal pathways of the central complex, for example, the expression pattern of neuromodulators only reaches maturity shortly before, or at hatching from the egg (Boyan et al. 2010; Boyan and Liu 2016) as may prove to be the case for the antennal system here.

## References

[CR1] Banzai K, Adachi T, Izumi S (2015). Comparative analyses of the cholinergic locus of ChAT and VAChT and its expression in the silkworm *Bombyx mori*. Comp Biochem Physiol B.

[CR2] Bentley D, Keshishian H, Shankland M, Torian-Raymond A (1979). Quantitative staging of embryonic development of the locust, *Schistocerca nitens*. J Embryol Exp Morphol.

[CR3] Blagburn JM, Sattelle DB (1987). Presynaptic depolarization mediates presynaptic inhibition at a synapse between an identified mechanosensory neurone and giant intemeurone 3 in the first instar cockroach, *Periplaneta americana*. J Exp Biol.

[CR5] Boppana S, Kendall N, Akinrinsola O, White D, Patel K, Lawal H (2017). Immunolocalization of the vesicular acetylcholine transporter in larval and adult *Drosophila* neurons. Neurosci Lett.

[CR6] Boyan GS (1988). Presynaptic inhibition of identified wind-sensitive afferents in the cercal system of the locust. J Neurosci.

[CR7] Boyan GS, Ball EE (1993). The locust, *Drosophila*, and neuronal homology. Prog Neurobiol.

[CR9] Boyan GS, Liu Y (2016). Development of the neurochemical architecture of the central complex. Front Behav Neurosci.

[CR10] Boyan G, Williams L, Herbert Z (2010). Multipotent neuroblasts generate a biochemical neuroarchitecture in the central complex of the locust *Schistocerca gregaria*. Cell Tissue Res.

[CR12] Buchner E, Buchner S, Burg MG, Hofbauer A, Pak WL, Pollack I (1993). Histamine is a major mechanosensory neurotransmitter candidate in *Drosophila melanogaster*. Cell Tissue Res.

[CR13] Casares F, Mann RS (1998). Control of antennal versus leg development in *Drosophila*. Nature.

[CR14] Chapman RF (2002). Development of phenotypic differences in sensillum populations on the antennae of a locust, *Schistocerca americana*. J Morphol.

[CR15] Chapman RF, Greenwood M (1986). Changes in distribution and abundance of antennal sensilla during growth of *Locusta migratoria* L. (Orthoptera: Acrididae). Int J Insect Morphol Embryol.

[CR16] Deshpande S, Freyberg Z, Lawal HO, Krantz DE (2020). Vesicular neurotransmitter transporters in *Drosophila melanogaster*. BBA Biomembranes.

[CR17] Ehrhardt E, Kleele T, Boyan GS (2015). A method for immunolabeling neurons in intact cuticularized insect appendages. Dev Genes Evol.

[CR18] Ehrhardt EE, Graf P, Kleele T, Liu Y, Boyan GS (2016). Fates of identified pioneer cells in the developing antennal nervous system of the locust *Schistocerca gregaria*. Arthr Struct Dev.

[CR19] Fabian-Fine R, Anderson CM, Roush MA, Johnson JAG, Liu H, French AS, Torkkeli PH (2017). The distribution of cholinergic neurons and their co-localization with FMRFamide, in central and peripheral neurons of the spider *Cupiennius salei*. Cell Tissue Res.

[CR20] Geffard M, Vieillemaringe J, Heinrich-Rock A-M, Duris P (1985). Anti-acetylcholine antibodies and first immunocytochemical application in insect brain. Neurosci Lett.

[CR21] Gewecke M (1972). Bewegungsmechanismen und Gelenkrezeptoren der Antennen von *Locusta migratoria* L. Z Morph Tiere.

[CR22] Goodman CS, Spitzer NC, Sattelle DB, Hall LM, Hildebrand JG (1980). Embryonic development of neurotransmitter receptors in locusts. Receptors for neurotransmitters, hormones and pheromones in insects.

[CR23] Goodman CS, O'Shea M, McCaman R, Spitzer NC (1979). Embryonic development of identified neurons: temporal pattern of morphological and biochemical differentiation. Science.

[CR24] Heinrich R, Hedwig B, Elsner N (1997). Cholinergic activation of stridulatory behaviour in the locust *Omocestus viridulus*, (L.). J Exp Biol.

[CR25] Hermsen B, Stetzer E, Thees R, Heiermann R, Schrattenholz A, Ebbinghaus U, Kretschmer A, Methfessel C, Reinhardt S, Maelicke A (1998). Neuronal nicotinic receptors in the locust *Locusta migratoria*. Cloning and expression. J Biol Chem.

[CR26] Homberg U (2002). Neurotransmitters and neuropeptides in the brain of the locust. Microsc Res Tech.

[CR27] Jan LY, Jan YN (1982). Antibodies to horseradish-peroxidase as specific neuronal markers in *Drosophila* and locust embryos. Proc Natl Acad Sci USA.

[CR28] Jia H-G, Yamuy J, Sampogna S, Morales FR, Chase MH (2003). Colocalization of γ-aminobutyric acid and acetylcholine in neurons in the laterodorsal and pedunculopontine tegmental nuclei in the cat: a light and electron microscopic study. Brain Res.

[CR29] Kitamoto T, Wang W, Salvaterra PM (1998). Structure and organization of the *Drosophila* cholinergic locus. J Biol Chem.

[CR30] Knipper M, Strotmann J, Mädler U, Kahle C, Breer H (1989). Monoclonal antibodies against the high affinity choline transport system. Neurochem Int.

[CR31] Kunst M, Pförtner R, Aschenbrenner K, Heinrich R (2011). Neurochemical architecture of the central complex related to its function in the control of locust acoustic communication. PLoS ONE.

[CR32] Leitch B, Watkins BL, Burrows M (1993). Distribution of acetylcholine receptors in the central nervous system of adult locusts. J Comp Neurol.

[CR33] Leitinger G, Simmons PJ (2000). Cytochemical evidence that acetylcholine is a neurotransmitter of neurons that make excitatory and inhibitory outputs in the locust ocellar visual system. J Comp Neurol.

[CR34] Lutz EM, Tyrer NM (1987). Immunohistochemical localization of choline acetyltransferase in the central nervous system of the locust. Brain Res.

[CR35] Meier T, Reichert H (1991). Serially homologous development of the peripheral nervous system in the mouthparts of the locust. J Comp Neurol.

[CR36] Meier T, Chabaud F, Reichert H (1991). Homologous patterns in the embryonic development of the peripheral nervous system in the locust *Schistocerca gregaria* and the fly *Drosophila melanogaster*. Development.

[CR37] Nässel DR (2002). Neuropeptides in the nervous system of *Drosophila* and other insects: multiple roles as neuromodulators and neurohormones. Prog Neurobiol.

[CR38] Ochieng S, Hallberg E, Hansson B (1998). Fine structure and distribution of antennal sensilla of the desert locust, *Schistocerca gregaria* (Orthoptera: Acrididae). Cell Tissue Res.

[CR39] Persson MGS, Nässel DR (1999). Neuropeptides in insect sensory neurones: tachykinin-, FMRFamide- and allatotropin-related peptides in terminals of locust thoracic sensory afferents. Brain Res.

[CR40] Python F, Stocker RF (2002). Immunoreactivity against choline acetyltransferase, γ-aminobutyric acid, histamine, octopamine, and serotonin in the larval chemosensory system of *Drosophila melanogaster*. J Comp Neurol.

[CR41] Rind FC, Leitinger G (2000). Immunocytochemical evidence that collision sensing neurons in the locust visual system contain acetylcholine. J Comp Neurol.

[CR42] Rind FC, Simmons PJ (1998). Local circuit for the computation of object approach by an identified visual neuron in the locust. J Comp Neurol.

[CR43] Salvaterra PM, Kitamoto T (2001). *Drosophila* cholinergic neurons and processes visualized with Gal4/UAS-GFP. Gene Expr Patterns.

[CR44] Sanes JR, Hildebrand JG (1976). Acetylcholine and its metabolic enzymes in developing antennae of the moth, *Manduca sexta*. Dev Biol.

[CR45] Sanes JR, Prescott DJ, Hildebrand JG (1977). Cholinergic neurochemical development of normal and deafferented antennal lobes during metamorphosis of the moth, *Manduca sexta*. Brain Res.

[CR46] Showell SS, Martinez Y, Gondolfo S, Boppana S, Lawal HO (2020). Overexpression of the vesicular acetylcholine transporter disrupts cognitive performance and causes age-dependent locomotion decline in *Drosophila*. Mol Cell Neurosci.

[CR47] Slifer EH, Prestage JJ, Beams HW (1959). The chemoreceptors and other sense organs on the antennal flagellum of the locust (Orthoptera; Acridiadae). J Morphol.

[CR48] Song HJ, Ming GL, Fon E, Bellocchio E, Edwards RH, Poo MM (1997). Expression of a putative vesicular acetylcholine transporter facilitates quantal transmitter packaging. Neuron.

[CR49] Soustelle L, Besson MT, Rival T, Birman S (2002). Terminal glial differentiation involves regulated expression of the excitatory amino acid transporters in the *Drosophila* embryonic CNS. Dev Biol.

[CR50] Stevenson PA, Kutsch W (1986). Basic circuitry of an adult-specific motor program completed with embryogenesis. Naturwissenschaften.

[CR51] Thompson KJ, Siegler MVS (1991). Anatomy and physiology of spiking local and intersegmental interneurons in the median neuroblast lineage of the locust. J Comp Neurol.

[CR52] Walker R, Holden-Dye L (1991). Evolutionary aspects of transmitter molecules, their receptors and channels. Parasitology.

[CR53] Weber M, Kössl M, Volknandt W, Seyfarth EA (2005) Acetylcholine is a transmitter candidate in sensory neurons of the bushcricket ear (*Mecapoda elongata*). In: Proceedings, 6th meeting German neuroscience society:Abstract 94A

[CR54] Yasuyama K, Salvaterra PM (1999). Localization of choline acetyltransferase-expression neurons in *Drosophila *nervous system. Microsc Res Tech.

